# Australian Aboriginal Birth Cohort study: follow-up processes at 20 years

**DOI:** 10.1186/1472-698X-9-23

**Published:** 2009-09-24

**Authors:** Susan Sayers, Gurmeet Singh, Dorothy Mackerras, Megan Lawrance, Wendy Gunthorpe, Lisa Jamieson, Belinda Davison, Kobi Schutz, Joseph Fitz

**Affiliations:** 1 Menzies School of Health Research, Institute of Advanced Studies, Charles Darwin University, Darwin, NT Australia; 2 Food Standards Australia New Zealand, Barton, ACT, Australia; 3 Australian Research Centre for Population Oral Health, University of Adelaide SA, Australia

## Abstract

**Background:**

In 1987, a prospective study of an Australian Aboriginal Birth Cohort was established focusing on the relationships of fetal and childhood growth with the risk of chronic adult disease. However as the study is being conducted in a highly marginalized population it is also an important resource for cross-sectional descriptive and analytical studies. The aim of this paper is to describe the processes of the third follow up which was conducted 20 years after recruitment at birth.

**Methods:**

Progressive steps in a multiphase protocol were used for tracing, with modifications for the expected rural or urban location of the participants.

**Results:**

Of the original 686 cohort participants recruited 68 were untraced and 27 were known to have died. Of the 591 available for examination 122 were not examined; 11 of these were refusals and the remainder were not seen for logistical reasons relating to inclement weather, mobility of participants and single participants living in very remote locations.

**Conclusion:**

The high retention rate of this follow-up 20 years after birth recruitment is a testament to the development of successful multiphase protocols aimed at overcoming the challenges of tracing a cohort over a widespread remote area and also to the perseverance of the study personnel. We also interpret the high retention rate as a reflection of the good will of the wider Aboriginal community towards this study and that researchers interactions with the community were positive. The continued follow-up of this life course study now seems feasible and there are plans to trace and reexamine the cohort at age 25 years.

## Background

The hypothesis on developmental origins of health and disease which relates growth of intra-uterine and early life to the risk of chronic disease in adult life may be particularly relevant to the Aboriginal peoples of the Northern Territory (NT). Reports mainly from developed populations relate low birth weight (LBW) and fetal growth restriction (FGR) to the risk of cardiovascular disease, type 2 diabetes and hypertension in adult life, particularly if rapid catch-up growth has occurred [1]. Currently for NT Aboriginal people, LBW rates are double those of the non-Aboriginal NT population [2] and more LBW babies are surviving into adult life as infant mortality rates for Aboriginal people have markedly improved over the last decades [3,4]. At the same time the rates of the chronic non-communicable adult diseases of cardiovascular disease, hypertension, type 2 diabetes and end stage renal disease are high [4-6] and as a result, life expectancies are similar to those for developing populations [4,7].

We therefore postulated that the improved survival of the LBW and FGR babies may be contributing to the rising rates of chronic diseases seen in the Aboriginal population. In 1987, a prospective study of an Australian Aboriginal Birth Cohort was established focusing on the relationships of fetal and childhood growth outcomes with the risk of chronic adult disease. However the study is also an important resource for cross-sectional descriptive and analytical studies as it is being conducted in a highly marginalized population of Aboriginal youth.

The recruitment and a previous follow-up of this cohort have already been described in detail [8]. In brief, 686 out of 1238 Aboriginal babies born at the Royal Darwin Hospital (RDH) between January 1987 and March 1990 were recruited. Although the participants were not randomly selected there were no differences for mean birth weights, gender ratio and birth weight frequency between those recruited and those not recruited [8]. Babies classified as below the 10^th ^percentile for gestational age were used as a surrogate for FGR babies. Using a post-natal estimation of gestational age and an Australian birth weight for gestational age reference contemporary with the recruitment interval, 27% of routine births were FGR instead of the expected 2.5% [8]. Cohort participants were later followed up in over 70 locations in northern Australia at a mean age of 11.4 years from December 1998 to January 2001[8]. At this time, overall growth outcomes for the cohort participants were poor, and compared with non-FGR children FGR children were shorter, lighter and had smaller head circumferences [9]. Furthermore, in this population of children with poor growth outcomes no inverse relationships of biomarkers of chronic disease with birth weight were present apart from with blood pressure [10]. Given the high prevalence of overweight and obesity seen in Aboriginal adults [4] these relationships may change with the development of adult obesity. Therefore we are keen to continue to follow these cohort participants to see if the relationships of biomarkers with birth weight change if and when the onset of overweight occurs. The information gained from this study will help determine the timing and forms of interventions to decrease the risk of chronic adult disease in this population.

Hence a further follow-up was conducted 20 years after the initial recruitment, with a high retention rate of the participants essential to produce valid outcomes. In this follow-up retention was highly dependent on the success of the processes used in tracking and maintaining the interest and co-operation of the now young adult participants.

### Aim

To describe the processes of a follow up of young men and women of the Aboriginal Birth Cohort conducted December 2005-January 2008.

## Methods

### Subjects

Aboriginal young adults belonging to a prospective longitudinal study of a birth cohort.

### Setting

**Figure 1:**
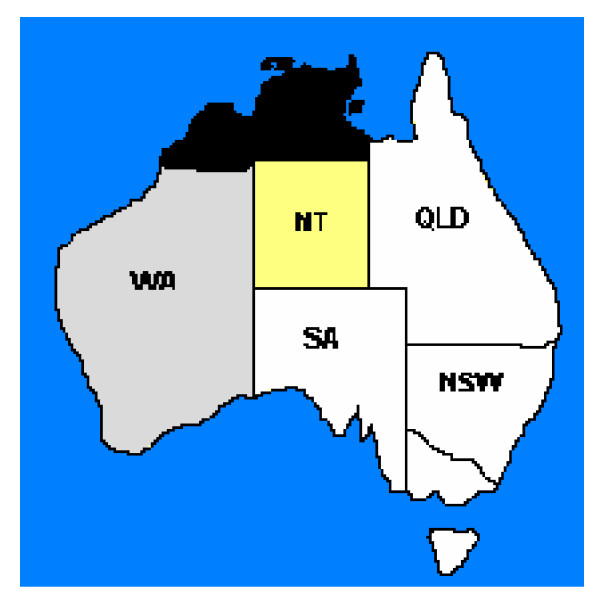
**Map of Australia showing residential area of participants at recruitment and follow-up: Aboriginal Birth Cohort 1987-2000**.

The catchment area was the geographic health region served by RDH. The region includes Darwin, the capital of the NT. and rural and remote towns and communities across the northern part of the NT and Western Australia. This sparsely populated area (0.2/km^2^) is approximately 2 million square kilometers (Figure. 1).

### Follow-up 2006-2008

Tracing was undertaken by one full time and one part time research assistant both of whom had no previous tracing experience. Progressive steps in a multiphase protocol, adapted from the previous follow-up, were used [8]. These steps were modified depending on whether the participants were thought to live in rural or urban locations (Table 1). A central electronic spreadsheet was used to log progress and follow-up leads. Weekly team meetings were conducted to coordinate tracking and follow-up efforts.

**Table 1:** Multiphase tracing protocol: Aboriginal Birth Cohort Study 2006-2008

Phase	Urban	Target rural community
**1**	Phone call and/or letter to last known address	List of names from community and wider area shown to key community people with request for follow-up information

**2**	Urban list reviewed face to face with local Aboriginal workers and lists compared with online phone directory, computerized electoral roll and local school rolls.	Local community workers, friends, family members other cohort participants shown list when research team visited
	Remaining names checked with urban Aboriginal communities and dedicated Aboriginal urban services	

**3**	Remaining names checked with hostels, churches, sporting clubs, corrective services	Remaining names shown again to key people

**4**	Each research team member given three untraced participants to check all avenues till no further leads found	

### Preparation

In preparation for follow-up, a manual audit of hospital medical records was undertaken in order to update addresses and communities recorded in the previous follow-up. Publicity for the study was generated through the local newspaper, radio stations, NT Departments of Health and Families and Education and Training, RDH Aboriginal liaison officers and the distribution of posters to community health clinics and an Aboriginal Health Worker conference. Electronic matches of names were undertaken with NT and Western Australian Death Registries to prevent distress to friends and family by inquiring about participants who had died.

### Tracing first phase

Initially all names were sorted by last known address. Lists of participants known to be living in specific communities and town locations were made. To preserve participant privacy, hospital registration number, gender, date of birth and name of the mother were shown to clinic staff only and withheld from other possible informants. A phone call was made to the senior worker of the community health clinic where participants were thought to currently live. The study was explained and staff cooperation sought. A list of participants last known to be in that community was then shown to a community health clinic worker with a request for information about participants' current whereabouts and any pertinent information for locating the subjects. In bigger communities, the clinic was also asked for contact details of local Aboriginal people who might be willing to undertake casual employment, tracing and locating participants in their community.

For urban participants, the last known address was contacted by phone or letter. If there was no response, the address was visited in person.

### Tracing second phase

For rural participants, information on participants' whereabouts was obtained from local community workers, next of kin, friends and other cohort members. Using this information, participants who were traced but not yet examined could be added to other community lists if necessary. It was not uncommon for a participant to be listed in more than one community.

For the urban participants, if phone, letter and personal visit to the address were all unsuccessful, then the names of participants thought to live in the urban area were reviewed on a one-to-one basis with local Aboriginal people known to the research team and Aboriginal liaison officers and interpreters from RDH. This list was also compared with an online telephone directory, the local electoral roll and the local high schools rolls. The remaining names of untraced participants were shown to individual workers of local Aboriginal urban residential communities and dedicated Aboriginal health services within Darwin.

### Tracing third phase

The names of the remaining untraced rural participants were shown to clinic and council members of the communities during the second visits.

The names of the remaining untraced urban participants were manually checked by researchers against names associated with the local Aboriginal hostels, sporting clubs, corrective services, churches, the Aboriginal Corporation of the Larrakia Nation (who are the traditional custodians of the land and water around Darwin) and The Northern Land Council (representing the traditional landowners and Aboriginal people in the Top End of the NT).

### Tracing fourth phase

Team members were each given up to three untraced participants to focus final tracing efforts until no further leads could be found. Finally in late 2007 electronic matches of the names of untraced participants were again undertaken with NT and Western Australian Death Registries.

### Data collection

Follow-up examinations commenced in December 2005 and continued till January 2008. The main follow-up team consisted of a pediatrician, a dentist, a psychologist, a male Aboriginal research assistant and full and part time research assistants. To accommodate team absences, team members had training in a number of procedures including venepuncture. However the ultrasound and dental assessments required the skills of specialists in these areas.

For urban participants, preparations for a group to be examined commenced about 2 weeks in advance. The research team remained flexible about places and times of assessments, although the clinic areas at RDH were frequently used. Telephone reminders were attempted prior to the assessment and transport to the assessment site was provided.

For the rural participants, liaison with the target community commenced 2-3 months prior to a planned visit. In contrast to the previous 11 year follow-up which was conducted mainly in community schools, we did not use these for the current follow-up as it was anticipated that few participants would be attending school. The current examinations were predominantly undertaken in spaces associated with the community health centers and also community halls, council spaces, school rooms and private spaces. A primary criterion for selecting a location was the availability of electrical power points for the equipment and toilets.

Table 2 shows the range of data collected in the current follow-up. The procedures took approximately 2 hours. Because participants may have been unwilling to spend this much time with the team, the assessments were prioritized and this was known to all researchers. Height, weight and a blood sample had the highest priority because not only did these data cover growth and the greatest number of biomarkers for chronic disease but they had also been collected in the previous follow-up. The cognitive function test and the social and emotional well being questionnaire were given low priority as these were time consuming. Assessments were staggered so that the participants were occupied most of the time. Two photo albums of participants from the previous follow-up and the consenting participants from the current follow-up were popular with participants, families and friends who recognized themselves from years past.

**Table 2:** Data collection and source of information: Aboriginal Birth Cohort Study 2006-2008

Data collected	Source of information (method and instrument for blood tests)
**Anthropometric and nutritional**	
Date of assessment	
Height	Portable stadiometer
Weight	Tanita model TBF 521
Head, mid upper arm, waist hip circumferences	Tape measure
Serum folate	Immunoassay, Roche 170
Fe, transferrin, ferritin	Immuno-turbidimetric, Roche Modular
**Renal**	
Renal size and morphology	Ultrasonography
Random urinary albumin	Immuno-nephelometric, Beckman Immage
Random urinary creatinine	Jaffe reaction, Roche Modular
Random urinalysis	Urinary dipstick
**Metabolic and cardiovascular**	
Fasting plasma glucose, total cholesterol, HDL-C, triglycerides	Enzymatic colorimetric, Roche Modular
Apolipoprotein A-1, Apolipoprotein B	Immuno-turbidimetric, Roche Hitachi 917
Lipoprotein(a)	Immuno-nephelometric, Beckman Immage
Fasting plasma insulin	Immunoassay, AbbottAxsym
Haemoglobin A1C	HPLC, primus PDQ+
Blood pressure	Welch Allyn Lifesigns monitor
**Haematological and infection**	
Full blood count	Coulter Max M
C reactive protein	Immuno-turbidimetric Roche Modular
Impetigo, scabies, acanthosis, nigricars and fungal infection	Physical examination
**Dental**	
Questionnaire	Participant
Oral examination	Dental examination
**Non-invasive cardiovascular assessment**	
Carotid intimal thickness measure	Portable Sonoheart Elite system and L38 10-5 linear transducer
Heart rate variability	ECG
Digital pulse volume	Pulse Trace
**Iodine status**	
Thryoid stimulating hormone	Immunoassay Roche E170
Thyroid size and morphology	Ultrasonography
Random urinary iodine concentration	Colorimetry, Sandell-Kolthoff reaction
**Hepatitis B immunization status**	
Hepatitis B virus serology	Hepatitis B virus surface antigen, Hepatitis B virus core antibody
**Emotional well being**	
Depression, suicide, anxiety and resilience	Participant-"Strong Souls" questionnaire
**Cognitive function**	
Memory and reaction time test	Cogstate-Computer Based Program
**Life style**	
Age commenced and amount of tobacco and cannabis smoking, alcohol consumption and petrol sniffing Soft drink consumption and daily exercise	Participant-Questionnaire
**Socio-economic status**	
Education, employment, household size and car ownership	Participant-Questionnaire
**Muscle strength**	
Grip strength	Dynamometer

### Management of biological samples

#### Specimen collection

A local anesthetic cream (EMLA) was applied and blood drawn 30-40 minutes after this application. Tubes for serum and plasma collection were centrifuged post clotting as soon as possible, almost all within 2 hours of collection. The serum and plasma were decanted into labeled cryo-tubes and frozen. Blood was decanted from the EDTA tube and frozen as whole blood for estimation of HbA1c. A random urine specimen was collected. This was decanted into appropriate tubes and frozen.

Participants received food and drinks after the venesection whether they had been fasting or not.

#### Specimen transportation and storage

Only the full blood count was analyzed at the local Darwin laboratory. For this, an EDTA specimen was ice packed in an insulated carrier. Depending on the collection locality and which was expected to be faster to the laboratory in Darwin, the specimen was transported by the Community Care Centre's normal courier service or with the study team.

For the other analyses completed in laboratories up to 3,000 km away, frozen serum, plasma and urine samples were transported in a cryogenic transport vessel (dry shipper) which maintained the temperature at -80C. The dry shipper accompanied the study team on their return to Darwin either via car or by aircraft in accordance with International Air Travel Association (IATA) guidelines. All researchers were trained in the regulations associated with transporting biological specimens including special care about packing and labeling. The frozen specimens were stored in the Menzies School of Health Research laboratory freezers at -80C. Specimens were then transported on dry ice in batches to the testing laboratories.

### Subsidiary studies

There were two specific subsidiary studies in this young adult follow-up.

#### Hepatitis B immunity

The participants of this cohort were among the first in Australia to receive Hepatitis B vaccination because of the greater risk of hepatitis B viral infection (HBV) in Aboriginal communities. A part of the serum sample was used to estimate the concentrations of hepatitis B surface antibody and hepatitis core antibody. This allows the current prevalence of HBV infection and long term (>15 years) persistence of HBV antibody to be reported for the first time in a hard to reach population of young Aboriginal men and women.

#### Iodine status

The 2004 National Iodine Nutrition Survey, which measured urinary iodine and thyroid volume in school-children, included mainland states of Australia but for logistic reasons excluded the NT [11]. Indigenous people were surveyed only as part of the general population. The spot urine sample collected to assess renal health in this current follow-up was also suitable for urinary iodine analysis. This study will provide original prevalence data about iodine status for a previously unstudied population group.

#### Analysis

The RDH where the participants were recruited, functions as both the hospital for all routine births from the local Darwin Health Region and a tertiary referral centre for high risk births from the adjacent health regions. Hence the total cohort does not reflect a particular geographic area. However those from the local health region are likely to be representative of that geographic area, so when we report prevalence data we restrict the analyses to participants living in the Darwin Health Region [12] but include all subjects when conducting longitudinal cohort analysis [13].

#### Ethics

The study was approved by Human Research Ethics Committee (HREC) of NT Department of Health & Community Services and Menzies School of Health Research, including the Aboriginal Ethical Sub-committee which has the power of veto. Study approval was also obtained from the Western Australian Aboriginal Health Information and Ethics Committee.

For each rural/remote Aboriginal Community, consultation was undertaken about the study and approval sought from the Community Council for a research team visit. The signed Council approval was submitted to the HREC before the first visit to a particular community was approved.

To organise the consent process efficiently, explanations were given to groups of up to three potential study participants. An information sheet (in English) was provided and explained by a researcher using visual aids. An attempt was made to gender match the researchers obtaining consent with participants. If a gender match was not possible participants were asked if they felt comfortable speaking to a study team member of the opposite gender. If they agreed, a female researcher sometimes dealt with young men but very rarely vice versa. We used a structured consent form, where participants consented or refused each individual procedure, rather than an overall consent form. As this allowed choices of procedures to be made and individual specific procedures to be refused we hoped that this would decrease the outright refusal to participate in the study. Participants also consented, or refused, to allow their photographs to be used for various purposes.

As the participants were expected to be aged 16-19 years and some were likely to have their own children, the "mature minor" rule [14] was adopted in line with Guidelines for the General Consent of the RDH. The mature minor rule allows a child (under 18 years of age) with sufficient intellectual development and capacity to understand the nature and effect of the relevant treatment to be capable of giving consent. The age for this is not specified in the NT but in New South Wales and South Australia, it is identified as 14 and 16 years respectively. Available parents and careers were informed about the study and were encouraged to be present, but the participant themselves were the signatories.

Responses to identified problems were organised according to the urban rural setting (Table 3). Acute medical problems requiring immediate care were referred directly to the rural community clinic or the nominated health carer for urban participants; less acute conditions were routinely referred to health carers. Participants were given print-outs of their body fat percentage and explanations given to them in the context of their current height and weight.

**Table 3:** Team responses to identified problem: Aboriginal Birth Cohort Study 2006-2008

Identified problem	Response	
	Urban	Rural
Medical condition	Nominated GP or dedicated Aboriginal clinic notified	Community health clinic notified

Dental condition	Referred to urban dental service or private clinic	Referred to community dental service

Self harm risk	Referred to participants preferred urban mental health service	Notified nominated support person.
		Notified community health clinic for referral to mental health service and provided copies of questionnaires

Non- immune hepatitis B status(research officer employed specifically for this)	HBV vaccine booster doses	HBV vaccine booster doses

Procedures for young people identified as risk for self harm in the social and emotional well-being questionnaire required special consideration. Consultation was undertaken with local mental health professionals as to the availability of appropriate services if participants were detected in a state of crisis or immediate self harm. Researchers were reassured that community clinics were equipped to deal with acute mental health problems by accessing expert advice from the Adolescent Mental Health Crisis Team in Darwin via phone or video link depending on the resources at the clinic.

If responses to the emotional-well being questions suggested a risk of self harm a staged procedure directed researchers to undertake a second questionnaire derived from the Applied Suicide Intervention Skills Training (ASIST) manual produced by LivingWorks Education Inc.[15]. A participant with confirmed risk of self harm was to be accompanied with written referral to the most senior health worker at the community health clinic. The senior health worker, an Aboriginal health worker or mental health worker and/or the participant's nominated confidant assessed the urgency in the short term and immediate consultation or later referral to appropriate mental health services was arranged.

#### Sense of belonging and incentives

We hoped to improve current and future response rates by fostering a sense of the participants belonging to a special group, tagged the "Clan Cohort", at all participant contacts. A logo was created, posters were developed with a colorful cartoon theme and a Clan Cohort website was established following the same colorful cartoon theme http://edison.menzies.edu.au/clancohort/index.htm The logo was used on all correspondence, posters, T-shirts worn by the research team and canvas bags (with pens, dental items, and wrist bands) given to the participants.

## Results

**Figure 2:**
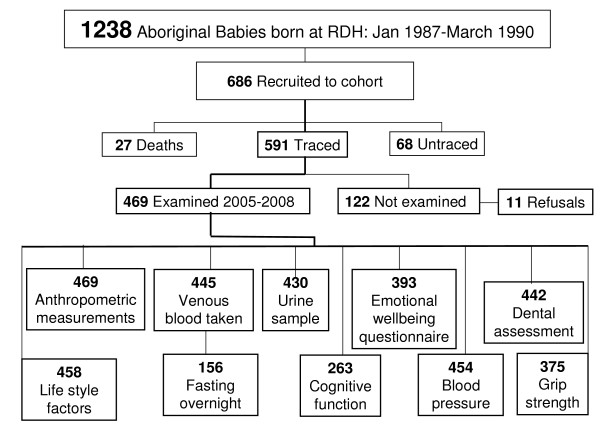
**Flow chart participants seen at follow-up December 2005 - January 2008**.

Of the 686 original participants recruited, 618 were traced of which 27 are known to of died. Of the 591 available for examination, 122 participants were not examined but only 11 refused outright and the remainder were not seen because of logistic reasons relating to inclement weather, mobility of participants and single participants living in very remote locations. Details are shown in Figure 2.

The number of known deaths had increased from 18 at the 11-year old follow-up to 27 at the current follow-up. These included 10 neonatal deaths and 10 traumatic deaths of which 4 were non-accidental

There were no significant differences between participants seen at this follow-up and those in the original cohort in regard to mean birth weight grams (3030 SD641 versus 3019 SD667, p = 0.9), gestational age weeks (38.7 SD1.96 versus 38.8 SD1.81, p = 0.4), gender ratio and proportions of LBW (17.8% versus 16%, p = 0.4), preterm (10.6% versus 10.5%, p = 0.9) and FGR (27.9% versus 27.5%, p = 0.9).

The mean age at this follow-up was 18.4 years; 50% were males, 14% were suburban residents of Darwin and the satellite town of Palmerston. Of the 469 seen, 114 had children, with young women (78/229) more likely to report children than the young men (36/229) (p < 0.001).

Although most participants consented to all the individual procedures on the structured consent form, the proportion of participants with actual measurements varied according to the procedures. 99% of consenting participants had both height and weight recorded and 94% had blood taken, 92% provided a urine sample, with 96% having blood pressure recorded and 83% had a digital pulse volume tracing taken. Over 90% of participants consented to do the cognitive study using computerized card sorting, but only 56% actually did this activity.

The emotional wellbeing questionnaire was completed by 83% of participants, of those almost half needed to do the second ASIST questionnaire, to check for risk of self harm. However after completion of the ASIST questionnaire no participants were found in a state of crisis or of immediate high risk and so the urgent referral protocols did not have to be activated.

### Costs

Costs of follow-up were dependent on the distance travelled to a locality, the number of potential participants to see and the number of participants actually seen per visit. Table 4 presents the estimated cost per participant for 5 team members visiting a remote community for 4 days to see a potential 40 participants and actually seeing 30. Costs associated with tracing, preparations for visit and salaries of team members are not included.

**Table 4:** Estimated minimum cost per participant for research team visit for 4 days to a community to see 40 participants and actually seeing 30: Aboriginal Birth Cohort Study 2006-2008

Item	Estimated cost Aus $
Aircraft travel $320 × 5 team members	1600
Accommodation $65 per night × 3 nights × 5	975
Subsistence allowance $60 per night × 3 nights × 5	900
Local vehicle hire $80 per day × 4 days	320
Fuel $50 per day × 4 days	200
Aboriginal research assistant 8 hour day × 4 days ($25 per hour)	800
Food for participants $8 × 40 participants	320
Total for 30 participants	5115

Cost per participant	170

## Discussion

We expected a higher attrition rate than experienced in the previous follow-up because the participants were older, unlikely to be enrolled in school, and more independent and mobile than they were at 11 years. As anticipated, the attrition rate rose in this follow-up of the study. However a retention rate of 71% is highly acceptable 20 years after recruitment, particularly considering the geographic, cultural and climatic challenges of following-up this cohort [8]. We interpret the 11 outright refusals and various partial refusals on the consent forms as an indication of the success of the structured and itemised informed consent process, as participants could pick and choose what testing items they were comfortable to perform and were not inhibited in indicating this information. While it is not possible to determine the role of the structured consent in achieving the high follow-up rate, we plan to use this again in future follow-ups.

Failure to examine traced participants was due to single participants living in very remote localities, aircraft cancellations due to weather conditions, local flooding and participant absence from a community at the time of scheduled research visits.

The urban dwellers were more difficult to trace and the fourth phase of the tracing protocol was frequently needed for these participants. We think the social networks, which assist with tracing, were more easily accessed by the researchers in the rural communities compared to the urban areas. This may have accounted for only 13% of participants seen at this follow-up being urban dwellers compared to the 21% at 11 years [8].

Because privacy is likely to be of greater concern for this age group than at a younger age, we reassured participants that they could always keep their clothes on, temporary screens were used and young men and women were managed separately. The equal representation of gender at this follow-up implies that young men and women were equally co-operative and suggests that the engagement of the male Aboriginal research assistant with the young men was positive and productive.

Participants with babies and young children were encouraged to bring them to the assessment which meant baby and child minding was needed while some procedures were underway.

Costs in this wave were higher than those in the previous follow-up due to sharp rises in fuel costs affecting both research team travel and transfer of biological samples to distant laboratories. Increased participant mobility also meant some bigger more distant communities required up to three visits. A larger number of team members were needed to accommodate the increased aspects being examined, salaries had also increased and more extensive testing of biological samples was done.

The two sub-studies conducted illustrate how a longitudinal study, particularly in a marginalized population, can contribute valuable information unrelated to the main purpose of the study. Both the Hepatitis B and Iodine sub-studies contribute to assessment and evaluation of national programs and illustrate that researchers might be able to fill data gaps opportunistically if they are aware of them.

## Conclusion

The high retention rate of this follow-up 20 years after birth recruitment implies the multiphase protocol used was successful and that researcher's interactions with the community were positive. We also interpret the high retention rate as a reflection of the good will of the wider Aboriginal community towards this study as well as possibly a perceived social or cultural expectation of the participants. Most cohort participants agreed to participate once they were traced. The members of the rural and remote communities remained interested and helpful and all the Aboriginal organizations approached for tracing were cooperative. Despite the costs and logistic challenges the continued follow-up of this life course study now seems feasible with plans to trace and reexamine the cohort at age 25 years. We anticipate that the high retention rates achieved in this follow-up can be replicated in the next follow-up.

As many contemporary life course studies relate to populations with low rates of LBW and FGR births the continued study of this cohort with high rates LBW and FGR is important considering the potential economic impact of improved birth weight on chronic disease outcomes in populations similar to this Aboriginal population [16].

## Competing interests

The authors declare that they have no competing interests.

## Authors' contributions

SS made contributions to conception and design, drafted the original manuscript and has been involved with revising the manuscript critically for important intellectual content.

GS made substantial contributions to conception and design, was responsible for the acquisition of data, and has been involved in revising the manuscript critically for important intellectual content. DM made substantial contributions to conception and design and has been have been involved in revising the manuscript critically for important intellectual content. ML made substantial contributions to acquisition of data, and has been involved in revising the manuscript critically for important intellectual content. LJ developed the oral health study, assisted with acquisition of data, and revising the manuscript. WG developed the social and emotional well being questionnaire and assisted with the acquisition of data and revising the manuscript. BD Assisted with acquisition of data and drafting and revising the manuscript. KS Assisted with acquisition of data and revising the manuscript. JF Assisted with acquisition of data and revising the manuscript. All authors read and approved the final version of the manuscript.

## Pre-publication history

The pre-publication history for this paper can be accessed here:

http://www.biomedcentral.com/1472-698X/9/23/prepub
